# Targeted Metabolomics in High Performance Sports: Differences between the Resting Metabolic Profile of Endurance- and Strength-Trained Athletes in Comparison with Sedentary Subjects over the Course of a Training Year

**DOI:** 10.3390/metabo13070833

**Published:** 2023-07-10

**Authors:** Mario Parstorfer, Gernot Poschet, Dorothea Kronsteiner, Kirsten Brüning, Birgit Friedmann-Bette

**Affiliations:** 1 Department of Sports Medicine, University Hospital Heidelberg, 69120 Heidelberg, Germany; birgit.friedmann-bette@med.uni-heidelberg.de; 2 Olympic Training Centre Rhine-Neckar, 69120 Heidelberg, Germany; kirsten.bruening@t-online.de; 3 Centre for Organismal Studies, Heidelberg University, 69120 Heidelberg, Germany; gernot.poschet@cos.uni-heidelberg.de; 4 Institute of Medical Biometry and Informatics, University of Heidelberg, 69120 Heidelberg, Germany; kronsteiner@imbi.uni-heidelberg.de

**Keywords:** athletes, phenotype, athlete metabolome, basal state, chronic adaptation, plasma

## Abstract

Little is known about the metabolic differences between endurance and strength athletes in comparison with sedentary subjects under controlled conditions and about variation of the metabolome throughout one year. We hypothesized that (1) the resting metabolic profile differs between sedentary subjects and athletes and between perennially endurance- and strength-trained athletes and (2) varies throughout one year of training. We performed quantitative, targeted metabolomics (Biocrates MxP^®^ Quant 500, Biocrates Life Sciences AG, Innsbruck, Austria) in plasma samples at rest in three groups of male adults, 12 strength-trained (weightlifters, 20 ± 3 years), 10 endurance-trained athletes (runners, 24 ± 3 years), and 12 sedentary subjects (25 ± 4 years) at the end of three training phases (regeneration, preparation, and competition) within one training year. Performance and anthropometric data showed significant (*p* < 0.05) differences between the groups. Metabolomic analysis revealed different resting metabolic profiles between the groups with acetylcarnitines, di- and triacylglycerols, and glycerophospho- and sphingolipids, as well as several amino acids as the most robust metabolites. Furthermore, we observed changes in free carnitine and 3-methylhistidine in strength-trained athletes throughout the training year. Regular endurance or strength training induces changes in the concentration of several metabolites associated with adaptations of the mitochondrial energy and glycolytic metabolism with concomitant changes in amino acid metabolism and cell signaling.

## 1. Introduction

Regular training performed for many years leads to the development of characteristic athletic phenotypes depending on the training content and the combination of intensity and volume, provided that the genetic profile is appropriate [1,2,3]. On one side of the exercise continuum, endurance training induces an increase in oxygen uptake, oxygen-transport capacity, and in oxidative metabolism with enhanced mitochondrial density in rather small muscles containing predominantly oxidative type I myofibers. These adaptations result in a greater resistance to fatigue and an enhanced endurance performance [4,5,6,7]. On the other side of the spectrum, resistance training leads to an increase in muscle mass with predominantly fast type II myofibers, favoring anaerobic and anabolic metabolism, and to an optimization of neuromuscular function [8,9], adaptations which enable maximal and fast strength development. The results of a few recent studies suggest that the adaptation to extreme forms of endurance or strength training leads to characteristic metabolic profiles which can be elucidated by applying metabolomics, allowing for the simultaneous analysis of low-molecular metabolic compounds [10,11,12,13].

Little information is available regarding long-term adaptation of the basal metabolome in chronically trained individuals, especially in basal state [11,14]. To the best of our knowledge, there is only one landmark study which investigated metabolic differences between athletes from different sport disciplines [12]. At the time when 191 athletes reported to anti-doping controls, blood samples were obtained for the measurement of 743 metabolites. The samples were collected as part of anti-doping controls in or out of competition with a lack of information about the athletes’ age, ethnicity, body mass, or their current training status, all factors with probably considerable effects on the individual metabolome. During the past years, research focused on changes of the basal metabolome of endurance-trained athletes and athletes of different sports in response to a single sport-specific session or multiple training weeks [15,16,17,18,19,20]. However, none of these studies investigated the effects of perennial training on the resting metabolome.

The aim of the present study was to investigate the differences in the resting plasma metabolome between endurance-trained athletes, strength-trained athletes, and sedentary subjects. A secondary objective was the assessment of changes in the metabolome over a one-year period involving different training and rest periods. We hypothesized that the resting plasma metabolome would exhibit distinct and significant characteristics between endurance-trained athletes, strength-trained athletes, and sedentary subjects. Furthermore, we expected significant changes in the metabolome to occur throughout the one-year study period, reflecting the influence of different training periods and regeneration phases. Therefore, the study includes several novel elements: (i) a comparison of the plasma metabolome of the physiological extremes under controlled conditions in (ii) a longitudinal study design throughout different training and rest periods in high-performance sports applying (iii) targeted, quantitative ultra-performance liquid chromatography tandem mass spectrometry (UPLC-MS/MS) using the MxP Quant 500 kit (Biocrates Life Sciences AG, Innsbruck, Austria).

## 2. Materials and Methods

### 2.1. Participants

Three groups of male adults, strength-trained athletes (ST; *n* = 12, age: 20.2 ± 2.6 years, mass: 80.3 ± 13.0 kg, height: 175.0 ± 8.7 cm, BMI: 26.0 ± 2.5 kg/m^2^, body fat: 10.6 ± 4.3%, VO_2_max: 42.5 ± 4.7 mL∙min^−1^∙kg^−1^), endurance-trained athletes (ET; *n* = 10, age: 24.0 ± 2.9 years, mass: 66.5 ± 9.2 kg, height: 176.8 ± 6.8 cm, BMI: 21.2 ± 2.1 kg∙m^−2^, body fat: 8.3 ± 1.4%, VO_2_max: 65.1 ± 4.8 mL∙min^−1^∙kg^−1^), and a group of sedentary subjects (CG; *n* = 12, aged 24.8 ± 4.2 years, mass: 81.9 ± 18.4 kg, height: 180.8 ± 8.8 cm, BMI: 25.2 ± 6.1 kg∙m^−2^, body fat: 14.9 ± 6.5%, VO_2_max: 41.2 ± 6.5 mL∙min^−1^∙kg^−1^) were investigated. The strength-trained group included weightlifters of the German Junior National Team (*n* = 8) and regional weightlifters (*n* = 4), practicing competitive sports for 8 ± 3 years at national and international levels. The endurance-trained group consisted of track and field runners with a specialization in middle- and long-distance running (800–10,000 m) as well as in marathon running, practicing competitive sports for 9 ± 4 years at regional and national levels. The control group included non-active, healthy participants who never underwent periodized training and who were unexperienced in endurance and resistance training. 

### 2.2. Experimental Design

**Figure 1:**
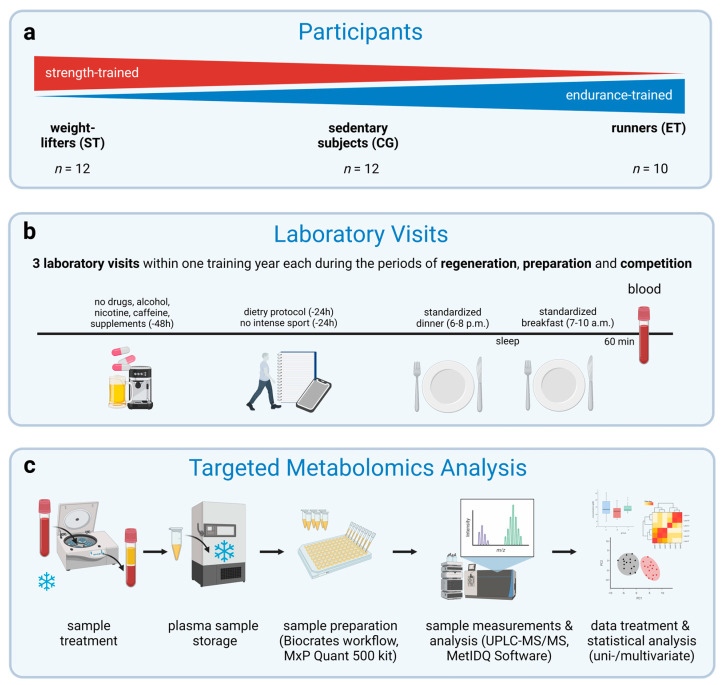
Schematic overview of the study design. (**a**) Chronically strength-trained (weightlifters, ST, 12 males) and endurance-trained athletes (runners, ET, 10 males) as well as healthy sedentary subjects (control group, CG, 12 males) were recruited. (**b**) Dietary control 24 h pre-laboratory visits. Subjects had to refrain from drugs, alcohol, nicotine, caffeine, and supplements 48 h before each visit. They had to avoid intense physical activity or training 24 h before each visit. All participants consumed a standardized dinner the day before each visit and a standardized breakfast on each visit. All participants performed three laboratory visits within one training year each during the periods of regeneration, preparation, and competition. Venous blood samples were taken from the forearm vein 60 min postprandial. (**c**) Sample treatment and analysis were performed by UPLC-MS/MS and MetIDQ Software according to Biocrates MxP Quant 500 kit workflow. Data treatment and statistical analysis were performed in the software R (version 4.0.3, R Core Team, Vienna, Austria) and MetaboAnalyst 4.0 (https://www.metaboanalyst.ca). The figure was created with BioRender.com.

Participants attended the laboratory on four separate occasions, the preliminary testing and three laboratory visits. Figure 1 provides a schematic representation of the experimental design. All participants received a medical examination before the preliminary testing. Participants were excluded if they presented evidence of musculoskeletal disorders (e.g., arthrosis, spondylarthrosis, spinal deformities), cardiovascular diseases (e.g., coronary heart disease, hypertension, cardiac arrythmias), acute or chronic diseases (e.g., infectious diseases, muscle diseases), or diseases related to obesity (e.g., type 2 diabetes mellitus, metabolic syndrome), as well as coagulation activity disorders and if they took regular medication.

### 2.3. Preliminary Testing

Body height and mass were measured using a standard stadiometer and a calibrated scale (Seca, Hamburg, Germany), respectively. The percentage of body fat was calculated from skinfold thickness measurements (Holtain, Crymych, UK) at 3 sites [21]. All participants performed an incremental exercise test to exhaustion either on a treadmill (ET and CG, ELG70, Woodway USA Inc., Waukesha, WI, USA) or on a cycle ergometer (ST, Excalibur Sport, Lode BV Medical Technology, Groningen, The Netherlands) to assess cardiorespiratory performance. Treadmill spiroergometry started with a 1-min warm-up at 4 km∙h^−1^. Then, running velocity (start at 6 km∙h^−1^, incline 1.5%) was increased by 2 km∙h^−1^ every 3 min until volitional exhaustion, followed by 5 min of recovery at 4 km∙h^−1^ and 5 min of passive rest. Cycling spiroergometry started at 50 W. The load was increased by 50 W every 3 min until volitional exhaustion, followed by 5 min of recovery with 50 W and 5 min of passive rest. Exhaustion was considered if at least two of the following criteria were met: high levels of blood lactate concentration (BLa; 8–10 mmol∙L^−1^); a plateau in oxygen uptake (VO_2_) despite increasing work rate; a respiratory exchange ratio (RER) above 1.1.

During each test, oxygen uptake (VO_2_), carbon dioxide release (VCO_2_), and ventilation (VE) were recorded with a breath-by-breath spirometry system (Geratherm Respiratory GmbH, Bad Kissingen, Germany) and the corresponding software Blue Cherry (version 1.3.0.5, Geratherm Respiratory GmbH, Bad Kissingen, Germany) using an individual adjusted face mask. Before each test, both sensors were calibrated with known gas concentrations and the flowmeter with a 3L-syringe according to the manufacturer’s instructions. Heart rate (HR) was recorded continuously with a 12-lead ECG using the Amedtec ECGpro Software (version 4.21.0, AMEDTEC Medizintechnik Aue GmbH, Aue, Germany) and self-adhesive electrodes in all three groups. Maximum oxygen uptake (VO_2max_) and RER_max_ were detected as the highest 30 s average values at the time of volitional exhaustion. If participants did not finish the entire increment, their maximal running velocity or cycling performance was linearly interpolated. 

### 2.4. Standardization

Participants were asked to refrain from any drugs (e.g., nicotine, alcohol, medication, caffeine) and soft drinks or special teas (green or black tea) 48 h before each laboratory visit. Nutritional supplementation (e.g., creatine, beetroot) was not allowed 48 h before each visit. All participants were asked to avoid intense physical activity and training 24 h before each visit. Participants were asked to not change their style of living (including mode of transportation) during the study. 

The basal metabolome was measured at the end of three characteristic training phases (for details see Tables S1 and S2): preparation (high volume and low intensity training), competition (regular competitions as well as low-volume, high-intensity training), and regeneration (no training at all or low-volume, low-intensity, unspecific training). All basal measurements were used for further analysis and declared together as the resting metabolic profile, independent of training phases. The results of the resting metabolic profile were part of a larger study where standardized endurance and strength tests were performed to investigate exercise-induced metabolic profile differences. The subjects of CG performed both the endurance and strength tests on different occasions. Therefore, their basal metabolome was determined twice. 

Due to the testing procedure and duration of the exercise tests and because of testing athletes in their competitive phase, it was not possible to test them in a fasted state. Therefore, all participants received nutrition counselling at the beginning of the study and a nutrition plan for a standardized dinner in the evening before each visit. They were served a standardized breakfast in the morning of each visit. All participants had to choose between three different meals in the evenings and in the mornings and stay with the same self-selected meal and size as well as the same mealtime during the study. Each meal consisted of an equal nutrient distribution (55–60% carbohydrates, 25–30% proteins, 13–17% fat). Water was consumed ad libitum. Compliance with the diet was confirmed with food diaries the day before the trials and a food photography method for lunch between 6 and 8 p.m. the evening before the trials [22]. Breakfast time was between 7 and 10 a.m. according to the participants’ schedule and consumed in a maximum of 20 min. To prevent possible bias related to circadian rhythms, all laboratory visits were performed at the same time of the day and sequence throughout the study [23]. 

### 2.5. Sample Handling

Venous blood samples (single 4.9 mL tube, EDTA, S-monovette, Sarstedt, Nümbrecht, Germany) were taken from the forearm vein of all participants in a seated position 60 min postprandial. After collection, the tube was immediately stored in crushed ice (4 °C) for no longer than two hours. Tubes were then centrifuged at 4000× *g* at 4 °C for 10 min. After separation, supernatant plasma was instantly aliquoted, snap frozen in liquid nitrogen, and stored at −80 °C until analysis.

### 2.6. Targeted Metabolomics Analysis

The Biocrates MxP^®^ Quant 500 kit (Biocrates Life Sciences AG, Innsbruck, Austria) can be used for analysis of up to 630 metabolites from 26 compound classes of widely different structures and polarities. Compound classes include lipids like acylcarnitines (Cx:y), hydroxylacylcarnitines [C(OH)x:y] and dicarboxylacylcarnitines (Cx:y-DC), lysophopatidylcholines, phosphatidylcholines, sphingomyelins (SMx:y) and sphingomyelin derivatives [SM(OH)x:y], ceramides and derivatives (cer-, hexcer-, hex2cer- and hex3cer-), cholesteryl esters, and diglycerides and triglycerides (the first fatty acid is counted individually, in the case of three fatty acids, the last two fatty acids are summed), which are all measured by FIA-MS/MS, as well as amino acids, amino acid-related compounds, bile acids, biogenic acids, biogenic amines, the sum of hexoses (H1), p-cresol sulfate, carboxylic acids, fatty acids, hormones and related metabolites (abscisic acid, cortisol, cortisone, dehydroepiandrosterone sulfate; DHEAS), indoles and derivatives (indole, 3-indoleacetic acid, 3-indolepropionic acid, indoxyl sulfate), xanthine and hypoxanthine, choline, trigonelline, and trimethylamine N-oxide (TMAO), which are determined by UPLC-MS/MS. 

In brief, 10 µL of human plasma were pipetted on a 96 well-plate containing internal standards and dried under a nitrogen stream using a positive pressure manifold (Waters). A total of 50 µL of a 5% phenyl isothiocyanate (PITC) solution was added to each well to derivatize amino acids and biogenic amines. After 1 h incubation at room temperature, the plate was dried again. To extract the metabolites, 300 µL 5 mM ammonium acetate in methanol was pipetted to each filter and incubated for 30 min. The extract was eluted into a new 96-well plate using positive pressure. For further LC-MS/MS analyses, 150 µL of the extract was diluted with an equal volume of water. For FIA-MS/MS analyses, 10 µL extract was diluted with 490 µL of FIA solvent (provided by Biocrates). After dilution, LC-MS/MS and FIA-MS/MS measurements were performed. For chromatographical separation, an UPLC I-class PLUS (Waters) system was used coupled to a SCIEX QTRAP 6500+ mass spectrometry system in electrospray ionization (ESI) mode. Data was generated using the Analyst (Sciex) software suite and transferred to the MetIDQ software (Biocrates Life Sciences AG), which was used for further data processing and analysis. All metabolites were identified using isotopically labeled internal standards and multiple reaction monitoring (MRM) using optimized MS conditions as provided by Biocrates. For quantification, either a seven-point calibration curve or one-point calibration was used depending on the metabolite class. Sample orders were randomized to ensure that the results obtained are not influenced by the order of analysis.

### 2.7. Statistical Analysis

All data preprocessing and analysis steps were performed using R (version 4.0.3, R Core Team, Vienna, Austria) [24]. Where appropriate, the web-based tool MetaboAnalyst 4.0 (https://www.metaboanalyst.ca) [25] was used. We used a multiple step procedure to ensure data quality. First, metabolites with more than 20% missing values (i.e., values lower than level of detection, <LOD) were removed from the data set [26]. Second, missing values were imputed using the k-nearest (k = 3) neighbor method [27,28]. In a third step, potential outliers were visually detected via principal component analysis (PCA). Samples far outside the 95% confidence interval were regarded as strong outliers. The final data matrix (367 metabolites and 34 samples) was used for further analysis in regard to group (ST, ET, CG) and training phase (PP, CP, RP). The control group was matched to both groups of athletes (matched to ET, CG-E; matched to ST, CG-S) separately and visited the laboratory twice at each training phase. When necessary, both control conditions were merged into a single control group (CG). 

Before analysis, all data were tested for normality of distribution using the Shapiro-Wilk procedure. Comparison of subject characteristics was performed using repeated measures analysis of variance (ANOVA). Features were log-transformed and analyzed by ANOVA with group (ST, ET, CG) and training phase (PP, CP, RP) in repeated measures design on training phase. We controlled for the effects of breakfast. Significant main effects were followed by pairwise comparisons with Bonferroni correction. For all statistical tests, the level of significance was set at 0.05. Data are presented as mean ± standard deviation (SD). Volcano plots with log_2_ fold-changes cut-off values (log_2_FC ≤ −1 & log_2_FC ≥ 1) were used to provide an overview of decreased and increased metabolite concentrations. To maximize the variance in the metabolic profiles between groups, training phase multivariate analysis was performed using MetaboAnalyst 4.0 (https://www.metaboanalyst.ca) [25]. Furthermore, multivariate analysis was used as single metabolites could be active in multiple pathways. Multivariate analysis was performed on log-transformed and auto-scaled data to correct for heteroscedasticity, to reduce skewness of the data, and to reduce mask effects [29]. Metabolites responsible for differences were identified using partial least squares discriminant analysis (PLS-DA) and variable importance in the projection (VIP) [30,31]. We used this to identify potential different metabolites between the groups and time points and to rank the metabolites according to their importance. VIP > 1.5 was considered sufficient for discrimination [32]. The quality of the PLS-DA model was estimated with a 10-fold cross validation method by goodness of fit (R^2^) and ability of prediction (Q^2^cum). The significance of class discrimination was assessed by permutation tests with 100 random permutation cycles due to the small sample size [33]. 

Uni- and multivariate statistical analysis were used to strengthen the results. Venn diagrams were used for simultaneous comparison of uni- and multivariate results where appropriate. Features were regarded as robust if univariate analysis revealed them as significant (*p* ≤ 0.05) with concomitant VIP ≥ 1.5 in multivariate analysis.

## 3. Results

### 3.1. Resting Metabolic Profile

**Figure 2:**
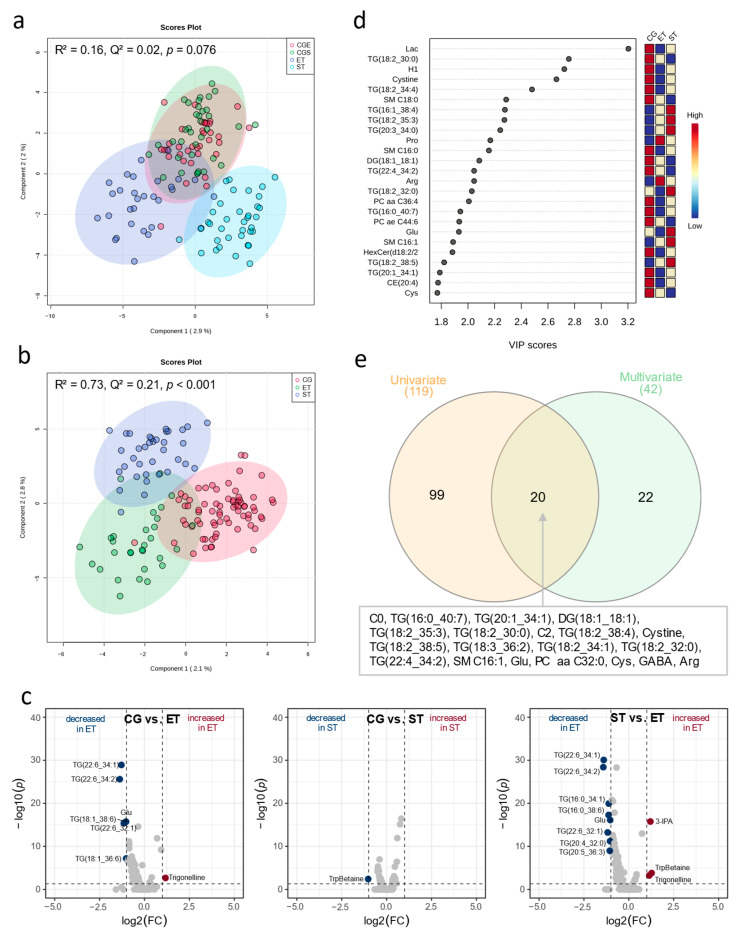
Univariate and multivariate analysis of resting metabolic profile of strength-trained (ST) and endurance-trained (ET) athletes and the control group (CG). (**a**) Score plot of partial least square discriminant analysis (PLS-DA) with two separated CG matched to both groups of athletes (control group matched to strength-trained athletes, CGS; control group matched to endurance-trained athletes, CGE). (**b**) and (**d**) PLS-DA with corresponding variable importance in projection (VIP) scores plot of the top 25 metabolites, where two control conditions were (CGS, CGE) merged to one CG. (**c**) Volcano plot depicting metabolomic diversity between the groups. Each point represents a metabolite. Dark blue and dark red indicate significantly (above dotted horizontal line) decreased or increased (fold change, FC ≥ 1.0, right and left side of dotted vertical lines) metabolites, grey indicates nondifferential metabolites. (**e**) Venn diagram for simultaneous comparison of uni- and multivariate analysis, showing 20 robust metabolites (see also Figure 2). Detailed data and metabolite abbreviations are summarized in Table S3.

**Figure 3:**
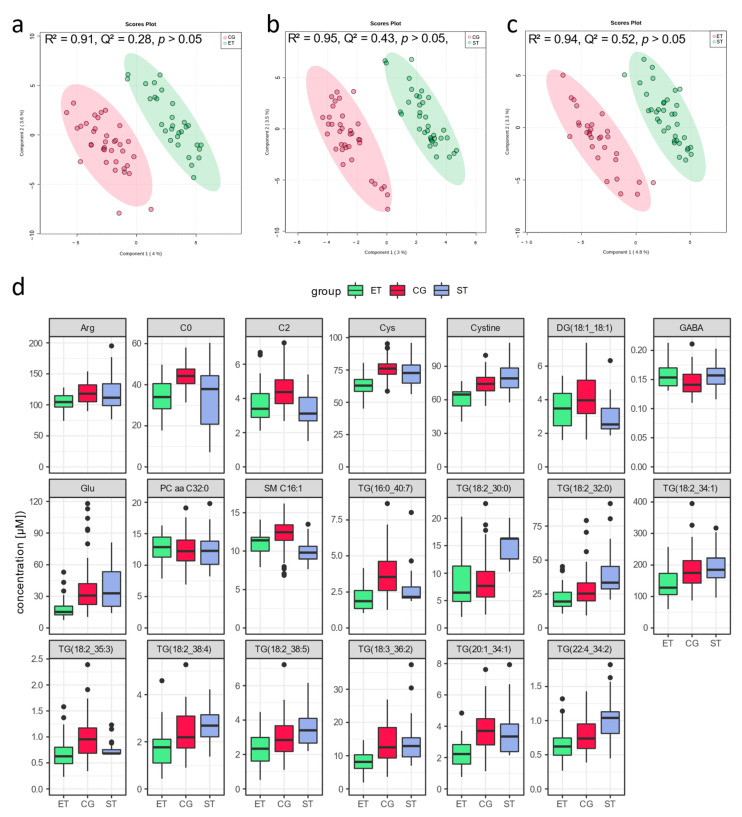
Multivariate and univariate analysis of resting metabolites of strength-trained (ST), endurance-trained (ET), and the corresponding control group (CG). (**a**) Score plot of partial least square discriminant analysis (PLS-DA) between CG and ET. (**b**) Score plot of PLS-DA between CG and ST. (**c**) Score plot of PLS-DA between ET and ST. (**d**) Boxplots (5–95% CI, median as black line) of the 20 robust metabolites representing the resting metabolite concentrations in each group (see also Figure 2e). Detailed data and metabolite abbreviations are summarized in Table S3.

PLS-DA revealed overlapping clusters of both tests in CG and a moderate visual discrimination between all groups (Figure 2a), especially when both control group conditions (endurance matched in CG, CG-E; strength matched in CG, CG-S) were merged into one single control group (Figure 2b,d). The corresponding model supports a robust discrimination with low predictive ability but statistical significance of model performance (R^2^ = 0.73, Q^2^ = 0.21, *p* < 0.001). PLS-DA showed a robust discrimination with moderate predictive ability between CG and ET (R^2^ = 0.91, Q^2^ = 0.28, *p* > 0.05) and CG and ST (R^2^ = 0.95, Q^2^ = 0.43, *p* > 0.05), as well as between ST and ET (R^2^ = 0.94, Q^2^ = 0.52, *p* > 0.05), but failed for statistical significance of model performance (Figure 3a–c). Univariate analysis with concomitant fold-change analysis of the baseline samples revealed significant differences in the resting metabolome of the three groups in several metabolites of five different compound classes: amino acids and amino acid-related metabolites, alkaloids, indole derivatives, and triglycerides (Figure 2c,d). In the univariate comparison of the resting metabolic profile in CG vs. ET, six metabolites were less abundant in ET: glutamate (Glu, FC = −1.0, *p* < 0.001) as well as the five triglycerides TG(22:6_34:1) [FC = −1.3, *p* < 0.001], TG(22:6_34:2) [FC = −1.4, *p* < 0.001], TG(18:1_38:6) [FC = −1.0, *p* < 0.001], TG(22:6_32:1) [FC = −1.2, *p* < 0.001], and TG(18:1_36:6) [FC = −1.0, *p* < 0.001], whereas the concentration of trigonelline (FC = 1.2, *p* < 0.001) was increased in ET. In the direct comparison of the resting metabolic profile in CG vs. ST, only the concentration of one metabolite was lower in ST: tryptophan betaine (TrpBetaine, FC = −1.0, *p* < 0.01). In the direct comparison of the resting metabolic profile in ST vs. ET, the concentration of three metabolites were increased in ET: 3-indolepropionic acid (3-IPA, FC = 1.2, *p* < 0.001), tryptophan betaine (FC = 1.3, *p* < 0.001), and trigonelline (FC = 1.0, *p* < 0.001). Eight metabolites were less abundant in ET: glutamate (Glu, FC = −1.0, *p* < 0.0001) as well as the seven triglycerides, TG(22:6_34:1) [FC = −1.4, *p* < 0.001], TG(22:6_34:2) [FC = −1.4, *p* < 0.001], TG(16:0_34:1) [FC = −1.1, *p* < 0.001], TG(16:0_38:6) [FC = −1.1, *p* < 0.001], TG(22:6_32:1) [FC = −1.2, *p* < 0.001], TG(20:4_32:0) [FC = −1.0, *p* < 0.001], and TG(20:5_36:3) [FC = −1.1, *p* < 0.001].

Detailed data is summarized in Table S3. The Venn diagram revealed 20 metabolites of eight different compound classes as robust (VIP > 1.5, *p* < 0.05) for group separation (Figure 2e): the two acylcarnitines carnitine (C0, VIP = 1.7, *p* < 0.001) and acetylcarnitine (C2, VIP = 1.6, *p* < 0.001), the amino acids and amino acid-related metabolites glutamine (Gln, VIP = 1.9, *p* < 0.05), arginine (Arg, VIP = 2.1, *p* < 0.001), cysteine (Cys, VIP = 1.8, *p* < 0.01), cystine (VIP = 2.7, *p* < 0.001), the biogenic amine γ-aminobutyrate (GABA, VIP = 1.7, *p* < 0.001), and lipid-related metabolites such as glycerophospholipids and sphingolipids, as well as di- and triglycerides: TG(16:0_40:7) [VIP = 1.9, *p* < 0.01], TG(20:1_34:1) [VIP = 1.8, *p* < 0.01], DG(18:1_18:1) [VIP = 2.1, *p* < 0.05], TG(18:2_35:3) [VIP = 2.3, *p* < 0.05], TG(18:2_30:0) [VIP = 2.8, *p* < 0.05], TG(18:2_38:4) [VIP = 1.5, *p* < 0.05], TG(18:2_38:5) [VIP = 1.8, *p* < 0.05], TG(18:3_36:2) [VIP = 1.6, *p* < 0.05], TG(18:2_34:1) [VIP = 1.5, *p* < 0.05], TG(18:2_32:0) [VIP = 2.0, *p* < 0.05], TG(22:4_34:2) [VIP = 2.1, *p* < 0.05], SM C16:1 [VIP = 1.9, *p* < 0.05], PC aa C32:0 [VIP = 1.7, *p* < 0.05].

### 3.2. Changes in the Metabolic Profile over One Year of Training

**Figure 4:**
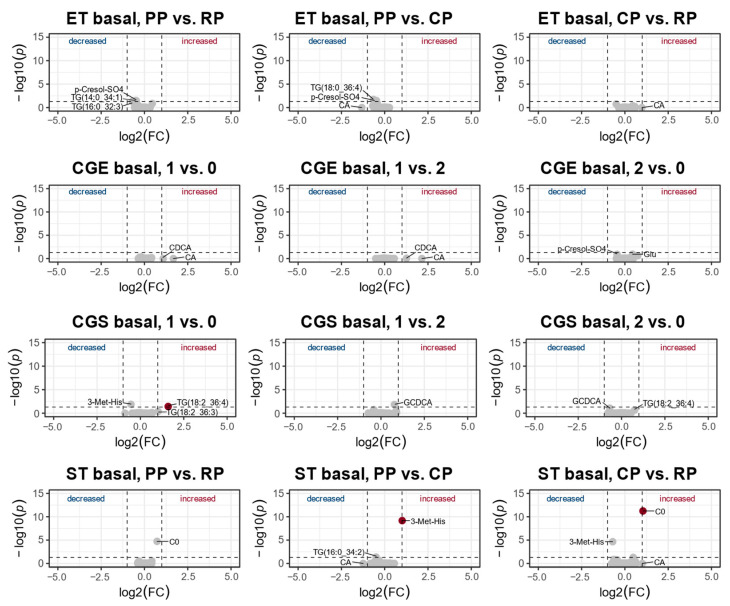
Analysis of the resting metabolic profile of endurance-trained (ET) and strength-trained (ST) athletes, as well as the control group matched to the endurance or strength group (CGE, CGS) over one year of training (CP, competition phase; PP, preparatory phase; RP, regeneration phase; 0, first visit; 1, second visit; 2, third visit). Volcano plot depicting metabolomic diversity in each group and training phase. Each point represents a metabolite. Dark blue and dark red indicate significantly (above dotted horizontal line) increased or decreased (fold change, FC ≥ 1.0, right and left side of dotted vertical lines) metabolites between training phases, and grey indicates nondifferential metabolites. Detailed data and metabolite abbreviations are summarized in Table S4.

PLS-DA revealed no valid models for the basal metabolic profile over one year of training. All models were highly overfitted (Q^2^ < 0) and failed for statistical significance; therefore, no score plots were reported (Figure 4). 

Univariate analysis revealed significant effects in basal concentration of four metabolites of three different compound classes over one year of training: acylcarnitines, amino acid-related metabolites, as well as triglycerides (Figure 4). In ST, basal concentration of carnitine (C0) was significantly increased in the competition phase compared to the regeneration [FC = 1.0, *p* < 0.001] and preparation phases [FC = 0.7, *p* < 0.001]. Furthermore 3-methylhistidine (3-Met-His) was significantly higher in preparation compared to the competition phase (FC = 1.0, *p* < 0.001). In CG-S, TG(18:2_36:4) [FC = 1.6, *p* < 0.001] as well as TG(18:0_36:4) [FC = 1.6, *p* < 0.001] were significantly higher in the second visit compared to the first visit (Figure 4). Detailed data is summarized in Table S4.

## 4. Discussion

The present study was designed to investigate characteristic metabolic adaptations to extreme forms of perennial endurance or strength training in whole body metabolism measured via blood metabolites [10,11,12,13]. To our best knowledge, for the first time, resting plasma metabolome was studied in strength-trained or endurance-trained athletes during one year in periods of preparing for competition, competing, or regenerating and compared with the findings in sedentary subjects. In summary, we observed a robust discrimination between the basal metabolome of the endurance- und strength-trained athletes as well as between these athletes and sedentary subjects, with mainly 20 metabolites from divergent compound classes. The most robust metabolites were free carnitine (C0) and acetylcarnitine (C2), different tri- (TGs) and diacylglycerols (DGs), phosphatidylcholines (PCs) and sphingolipids (SMs), the amino acids glutamate (Glu), arginine (Arg), cysteine (Cys), and cystine, as well as the biogenic amine γ-aminobutyrate (GABA). Our findings differed from the results reported by Schranner et al. [10], to our best knowledge the only other study on the effects of long-lasting endurance and strength training on the basal metabolome. However, the strength training performed by the body builders and sprinters investigated by Schranner et al. [10] certainly is a lot different from the strength training of the weight-lifters of the present study. 

With regard to the individual training phases, our analysis revealed two potential metabolites, free carnitine (C0) as well as 3-methylhistidine (3-Met-His), as training phase biomarkers for strength-trained athletes, while there was no endurance-specific set for endurance-trained athletes in resting condition.

All the observed differences between the differently trained athletes as well as between these athletes and the subjects of the control groups of the present study reflect systematic changes in the mitochondrial energy metabolism, e.g., glucose and fat metabolism, concomitant changes in amino acid metabolism, and cell signaling induced by specific, high volume and high intensity over several years.

### 4.1. Resting Metabolic Profile 

We observed lower resting concentrations in free carnitine (C0) and acetylated carnitine or acetylcarnitine (C2) in both groups of athletes compared to the sedentary subjects (Figure 2e and Figure 3d) with no different acylcarnitine to carnitine ratio between the groups. Compared to previous studies, the resting plasma concentrations of these metabolites were in the same range for all groups [10,34,35]. Acylcarnitines (Cx:y) are substrates produced for energy utilization (fat oxidation), as they are an activated form of fatty acid substrate which can be converted into acyl coenzyme A (CoA) without investing ATP [17,35,36]. Elevated resting levels of free carnitine (C0) and acetylcarnitine (C2) might indicate an increased availability of fatty acids in venous blood of the control subjects. It can be assumed that the lower plasma levels of free carnitine (C0) and acetylcarnitine (C2) in athletes are associated with an increased concentration of these carnitines in skeletal muscle where they are needed for the transport of long chain fatty acids from the cytosol into the mitochondria through the inner mitochondrial membrane [37]. Consecutively, this finding might suggest that also in strength-trained athletes, oxidative fat metabolism is of importance, probably during recovery from intense training sessions. In another study, changes in the mitochondrial transmembrane enzyme CPT1, which optimizes fatty acid transport from cytosol into the mitochondria, were reported [10]. If this is also the case for strength-trained athletes remains to be elucidated. In the study of Al-Khelaifi et al. [12], high endurance capacity was associated with reduced levels of two long-chain acylcarnitines (C18, C20:2) and, in accordance to the findings of the present investigation, increased short-chain acetylcarnitine (C2) levels. However, they did not investigate the basal metabolome in controlled conditions [12]. Schranner et al. [10] also observed higher concentrations of free carnitine (C0) and acetylcarnitine (C2) in controls compared to the group of athletes. In contrast to our findings, they additionally reported differences in medium- and long-chain acylcarnitines in rest. Due to strict quality requirements for the measured metabolites, medium- and long-chain fatty acids were declared as not valid for further analysis. In addition to the role of free carnitine (C0) in lipid metabolism described above, free carnitine (C0) is also capable of reducing post-exercise lactate (Lac) and prevent cellular damage [12,38]. It can thus be hypothesized that not only fatty acid oxidation and overall energy generation in rest, but also resting glucose metabolism of highly trained individuals, might be altered. 

In the present study, lactate (Lac) was strongly associated with group separation in multivariate analysis, with 1.3-fold higher values in the control subject compared to the group of athletes (Figure 2d, Table S3). However, lactate (Lac) was not observed as a robust metabolite after combining uni- and multivariate analysis (Figure 2e). Furthermore, the resting lactate (Lac) concentrations were all in the physiological ranges of 0.3–3.0 mM in blood for healthy individuals [39,40]. There is a steady lactate (Lac) homeostasis in a healthy environment with a transport between muscles, brain, heart, and gut. The reduction of pyruvate leads to the formation of lactate (Lac) under anaerobic and aerobic conditions [41]. There seems to be a variability in resting blood lactate (Lac) concentration which is related to overall metabolic capacity and risk for subsequent metabolic diseases due to a suppression of oxidative energy production capacity and a glycolysis-dependent ATP production [42,43]. The differences between control subjects and both groups of athletes can be explained by the regular activation of the lactate metabolism during training and competition in endurance- as well as in strength-trained athletes. Additionally, lactate was not found to be a discriminating metabolite between the groups of athletes and between the athletes and controls in the studies of Al-Khelaifi et al. and Schranner et al. [10,12]. 

In the present study, triacylglycerols (TGs) and diacylglycerols (DGs) were involved in the discrimination between the groups, with the lowest total triacylglycerols (TGs) and diacylglycerols (DGs) concentration in endurance-trained athletes (Figure 2c,d, Figure 3 and Figure 4, Table S3). The triacylglycerols (TGs) and diacylglycerols (DGs), which were less abundant in ET compared with CG and ST, mainly contained polyunsaturated fatty acids as linoleic acid (C18:2), arachidonic acid (C20:4), adrenic acid (C22:4), and docosahexaenoic acid (C22:6) (Figure 2c,d and Figure 3), but also the saturated palmitic acid (C16:0). The largest energy source in human metabolism is triacylglycerol stored within the skeletal muscle as an intramuscular substrate pool, readily built up or mobilized upon cellular energy needs [44]. In the study of Al-Khelaifi et al. [12], downregulation of diacylglycerols (DGs) containing linoleic acid (C18:2) and palmitic acid was associated with high endurance, whereas Schranner et al. [10] did not report differences in triacylglycerols (TGs) and diacylglycerols (DGs) concentrations. However, the findings in our group of endurance athletes suggest that long-lasting endurance training leads to changes in the resting energy metabolism by enhanced hydrolysis of triacylglycerols (TGs) and diacylglycerols (DGs) with an increased ability to oxidize fatty acids and activate lipolysis. 

There is an ongoing discussion that particularly triacylglycerols (TGs) and diacylglycerols (DGs) as well as sphingolipids (SM) are involved in signal transduction and promote insulin resistance in skeletal muscle [45,46]. Therefore, lower concentrations of several triacylglycerols (TGs) and diacylglycerols (DGs) as well as of the sphingomyelin (SM C16:1) in highly endurance-trained athletes is in accordance with increased insulin sensitivity in this group. Besides the either higher consumption or lower accumulation of sphingomyelin (SM C16:1) in both groups of athletes compared with the CG, the upregulation of phosphatidylcholines (PC aa C32:0) in both groups of athletes indicates differences in metabolic, neurological, and intracellular signaling processes in the endurance- and strength-trained athletes compared with sedentary subjects [47]. Sphingolipids (SM) and phosphatidylcholines (PCs) play an essential role in cell membranes and modulating cell functions, e.g., apoptosis, proliferation and differentiation, and inflammation, as well as lipid and glucose homeostasis [48,49]. Thus, the results of our study point to an increased membrane turnover and changes in glucose signaling in the group of athletes compared to the controls. The sum of all significant phosphatidylcholines (PCs) in a resting condition was 2.7-fold higher in athletes compared to non-athletes (Table S3). Different regulation of some phosphatidylcholines (PC ae C38:0, PC ae C36:5, PC ae C36:4, PC ae C38:6, PC ae C38:5, PC aa C36:6) in diverse groups of athletes and control subjects was also observed by Schranner et al. [10]. Higher serum concentrations of phosphatidylcholines (PCs) are associated with higher VO_2_max values, making PCs a potential biomarker for aerobic fitness effects and cardiovascular disease risk [50,51].

The amino acids cysteine (Cys), cystine, arginine (Arg), and glutamate (Glu) were under the top important metabolites for group discrimination (Figure 2d,e). We observed lower resting concentrations in the glucogenic amino acids glutamate (Glu) and arginine (Arg), as well as in the amino acids cysteine (Cys) and cystine, in ET compared with CG and ST (Figure 3d, Table S3). At rest, amino acids are used for gluconeogenesis and restoration of proteins and protein balance, as well as acid-based regulation [52]. Strength-trained athletes may have higher resting concentrations of amino acids due to changes in protein synthesis or turnover [10]. Decreased resting amino acid concentrations are associated with increased fitness levels, measured via VO_2_max, and increased fat oxidation rate during exercise [53]. Glutamate (Glu) is primarily found intracellularly and has limited ability to leave the cell, whereas glutamine (Gln) concentration is the highest extracellularly [54]. In the present study, the basal glutamate (Glu) concentration in endurance athletes was significantly decreased while the glutamine (Gln) concentration was similar in all the three groups. The glutamine-glutamate ratio is associated with better training condition and was higher in the endurance athletes than in the other two groups (data not shown) [55]. In addition, the group differences in glutamate (Glu) concentration may indicate a positive adaptive response in the glutamate-glutamine cycle of endurance-trained athletes probably due to greater utilization of glutamate (Glu) in muscle than to decreased production of glutamate (Glu). Glutamate (Glu) is an important source of α-ketoglutarate, an intermediate of the citrate cycle and thus essential for mitochondrial energy metabolism and high ATP synthesis, but is also associated as an important metabolite for alanine metabolism [56]. Additionally, removal of glutamate (Glu) is increased by the conversion of the inhibitory neurotransmitter γ-aminobutyrate (GABA) via glutamate decarboxylase [57]. GABA was highly important for resting group separation with the lowest concentration in controls compared to both groups of athletes (Figure 2e, Table S3). Elevated concentrations of γ-aminobutyrate (GABA) derivatives were reported by Al-Khelaifi et al. [12] and interpreted as a metabolic adaptation that might promote muscle growth. It might be speculated that γ-aminobutyrate (GABA) could be a marker for muscle growth in the resting condition during the preparatory training phase.

Univariate analysis detected tryptophan betaine (TrpBetaine) as well as 3-indoleproprionic acid (3-IPA) as significant metabolites (Figure 2c), whereas the combination with multivariate analysis considered them rather minor. For this reason, these two metabolites were not further discussed.

### 4.2. Changes of the Metabolic Profile over One Year of Training

We did not detect any differences in the basal metabolome of the endurance-trained athletes between the three training phases (Figure 4, Table S4). 

In strength-trained athletes, free carnitine (C0) concentrations were increased during the phases of preparation and competition compared with the phase of regeneration. As already discussed above, these changes in free carnitine (C0) might point to changes in cellular damage (probably an increase in cellular damage) and in lactate metabolism during the phases characterized by strenuous exercise. The concentration of amino acid 3-methylhistidine (3-Met-His) was increased during the preparatory phase compared with the phase of competition (Figure 4, Table S4). 3-methylhistidine (3-Met-His) is an essential component of the myofibrillar proteins acting in slow and fast-twitch muscle fibers and of heavy as well as light myosin chains of fast-twitch fibers. It is mainly present in skeletal muscle (91%) and to a lower degree in gastrointestinal tissues and skin, including connective tissues. Degradation of myofibrils ultimately leads to the release of 3-methylhistidine (3-Met-His), which could be quantitively measured in the urine and plasma [58,59]. Therefore, 3-methylhistidine (3-Met-His) is regarded as a biomarker for nucleotide breakdown and tissue homeostasis [60,61]. Apparently, the high-volume and low-intensity specific resistance training sessions during the preparatory period of strength-trained athletes might lead to high myofibrillar damage and z-disk disruption [62]. Changes in the level of 3-methylhistidine (3-Met-His) may suggest exercise-and training phase-specific changes in the rate of protein catabolism. In addition, one previous study revealed 3-methylhistidine (3-Met-His) as a fatigue marker [63].

### 4.3. Limitations

Standardization of training and testing sessions is a challenge in real-life studies, and it certainly is a reason for the few studies in the field of high-performance sports and for low subject numbers. However, low subject numbers could be the reason for missing significant differences between the three groups of our study. Therefore, validation studies with a larger number of participants would be valuable. Another source of weakness was the non-fasted state of the subjects. In an attempt to compensate for the non-fasted state, the subjects could choose one of three pre-meals with the same relative energy and macronutrients distribution as mentioned above. Chronic dietary and supplementation routines certainly had some influence on the basal metabolome. We found a high concentration of trigonelline only in some endurance athletes. Trigonelline is considered a dietary metabolite for coffee consumption without any biotransformation [64]. Day-to-day variability in metabolomics studies is minimal, but there are meal and collection time effects to be considered [23,65]. Future studies should be designed to avoid habitual caffeine consumption and dietary supplements for an even longer period of time before the measurements to reduce metabolome variability. Whether our results are transferable to female athletes remains to be elucidated.

## 5. Conclusions

In conclusion, we detected significant differences in the resting plasma metabolome between the endurance- and strength-trained athletes, as well as between the controls and both groups of athletes. Regular endurance or strength training seem to induce extreme changes in the concentration of several metabolites of athletes at rest. All differences between the basal metabolome of endurance- and strength-trained athletes and between these athletes and sedentary subjects were associated with the development of characteristic athletic phenotypes with concomitant systematic changes in mitochondrial energy metabolism, amino acid metabolism, and fatty acid oxidation, as well as cellular signaling. Furthermore, we found some strongly affected strength-specific metabolites between the training phases that were worth mentioning. 3-methylhistidine (3-Met-His) and free carnitine (C0) appeared as potential markers for protein catabolism and cellular damage in strength-trained athletes in certain training phases. 

## Data Availability

Data will be made available upon reasonable request by the corresponding author. Data is not publicly available due to privacy or ethical restrictions. The custom-made data analysis code for R is available from the corresponding author upon reasonable request.

## Supplementary Materials

The following supporting information can be downloaded at: https://www.mdpi.com/article/10.3390/metabo13070833/s1. Table S1: Details of the total training volume in each training phase of the strength-trained athletes (ST). Table S2: Details of the total training volume in each training phase of the endurance-trained athletes (ET). Table S3: Detailed data on metabolites between the groups. Table S4: Detailed data on metabolites between the training phases and groups.
